# Evaluating eye-tracking as a method for deception detection against the polygraph as deployed in practice

**DOI:** 10.1038/s41598-026-55344-9

**Published:** 2026-06-03

**Authors:** Weronika Celniak, Dominika Słapczyńska, Beata Jarszak, Piotr Augustyniak

**Affiliations:** 1 Department of Biocybernetics and Biomedical Engineering, AGH University of Krakow, Krakow, Poland; 2 Polish Forensic Association, Warsaw, Poland; 3 General Headquarters of the Border Guard, Warsaw, Poland

**Keywords:** Deception detection, Eye-tracking, Polygraph, Credibility assessment, Computational biology and bioinformatics, Engineering, Health care, Medical research, Neuroscience

## Abstract

Can credibility assessment be based on methods more reliable than those currently used? Traditional polygraph examinations, although widely employed, have well-documented limitations, motivating the search for alternative, noninvasive approaches. In this study, we developed an eye-tracking-based method to detect concealed memory traces by analyzing patterns of eye movements during a Concealed Information Test. Unlike previous research that examined eye movements in isolation, we directly compared the performance of the eye-tracking method with polygraph measurements within the same participants and under identical experimental conditions. Key features extracted from eye movements were used in a machine learning classifier to distinguish between deceptive and truthful responses. The results show that our eye-tracking-based test can effectively discriminate between deceptive and truthful individuals, achieving an accuracy of 94.9%. In comparison, polygraph examinations accurately classified 88.2% of participants under the same conditions, though five recordings had to be excluded because the polygraph expert could not reach a definitive conclusion. Finally, this study also considers the advantages and limitations of the two approaches, as well as ethical concerns related to measuring involuntary responses and using “black box” scoring algorithms.

## Introduction

The search for scientific methods that effectively discriminate deceptive from truthful individuals has a long and multidirectional history. One of the contemporary solutions, the polygraph, measures physiological responses associated with arousal, caused by stimuli of different meanings, as a function of probable deception or truthfulness. Despite the large number of surveys that have proved its accuracy, this tool remains a controversial method^1^.

Polygraphs are used in a variety of settings. In some countries such as Poland, Japan, and the Czech Republic, the result of the examination is particularly important because it is accepted as forensic evidence of the credibility of someone. In other countries, if not accepted in the courtroom, the polygraph can be used by the police during investigation work or by government agencies during the screening of their candidates for work. Taking into account the gravity of the results of polygraph testing in all of these applications, ensuring its accuracy is crucial.

The most important criticism towards polygraph is that certain tests (such as the Comparison Question Test) are not grounded in robust theory. Furthermore, polygraph examination can be disrupted by easy-to-learn countermeasures^2,3^. Another limitation of this tool is its susceptibility to high rates of false positives and false negatives, which can result in wrongful accusation of innocent individuals^4^ or incorrect dismissal of guilty individuals^5^. In addition, the multimodal nature of polygraph testing may lead to errors specific to each modality (e.g., issues related to electrode contact or subject movement). To address these challenges, ongoing research aims to develop new solutions to enhance the accuracy and reliability of lie detection methods.

With the aim of establishing an objective procedure, several modalities have been investigated, including fMRI^6^^7^ and EEG^8^, to measure brain activity associated with deception. This line of inquiry subsequently expanded to contactless measurement methods, such as remote photoplethysmography, voice analysis, and eye-tracking. Taking into account recent scientific findings, as well as the accessibility and practical applicability of the equipment, the latter approach has been of particular interest in our research.

Nowadays, eye-tracking technology can be employed to investigate cognitive activity associated with deception during the performance of various tasks. Scientific findings indicate that cognitive processes related to lying are multilayered and interconnected^9^^10^; however, eye-tracking enables the simultaneous observation of these interconnected layers during experiments, which is not possible with traditional polygraph examinations. To date, researchers have sought to analyze eye-related features during reading or the recognition of familiar items. The latter approach, based on the polygraph method known as the Concealed Information Test (CIT), remains a particularly promising area of research. It holds considerable potential for detecting concealed crime-related knowledge; however, further investigation is required to render it viable for widespread application in real-world scenarios.

Scientific evidence in the literature, as reported in^10^, suggests a direct link between deceptive behavior and oculomotor patterns, enabling the identification of individuals concealing memories with an accuracy of approximately 89%.

In parallel, a substantial body of research has explored the use of machine learning algorithms for analyzing physiological signals across various contexts, yielding promising results. Considering recent advancements, the integration of eye-tracking technology with machine learning tools offers the potential to develop artificial intelligence–based forensic systems for lie detection, capable of performing more complex tasks with higher accuracy and without the limitations associated with human-operated methods.

Currently, polygraph examinations in applied settings (i.e. police forensic laboratory, security agencies) require direct interaction between the examinee and a trained professional with extensive experience to conduct and interpret the examinations accurately. Such procedures are time-consuming and may be inconvenient for examinees, particularly if they lack confidence in the polygraph. Moreover, the accuracy of polygraph examinations can be affected by human factors, such as the examiner’s skill level. These challenges could be mitigated by the introduction of a scientifically valid, objective procedure that allows law enforcement or border control personnel, following relatively brief and straightforward training, to detect deception in real time.

To the best of our knowledge, no previous study has systematically compared the diagnostic accuracy of the Concealed Information Test (CIT) administered with both traditional polygraph-based physiological measures and eye-tracking indices within the same sample of participants under a controlled mock-crime paradigm. The present study was specifically designed to address this gap in the literature.

In this work, we hypothesize that oculomotor features associated with concealing guilty knowledge, when combined with machine learning, can be used to develop a novel contactless forensic tool. To test this hypothesis, we designed a mock crime scenario intended to closely mimic real-world settings. All participants were instructed to complete both polygraph testing and eye-tracking measurements in the same experimental environment to prove their innocence regarding the “crime” in question. Based on the collected oculomotor data, we evaluated whether a simple machine learning algorithm could be trained to discriminate concealed knowledge from these features.

Building on this, our second hypothesis proposed that eye-tracking would offer greater discriminative power in the CIT than traditional polygraph examinations. Polygraph tests were conducted under identical experimental conditions, allowing a direct comparison of the effectiveness of the two methods.

Finally, we compared the general utility of these approaches in detecting physiological indicators of deception. While the polygraph is a well-established tool with verified methodology, documented accuracy, and widely recognized limitations, methods based on eye-tracking remain under development, with ongoing research focused on determining the conditions necessary for a reliable forensic application. To our knowledge, this study is the first to directly compare the accuracy of a traditional polygraph and an eye-tracking method under spontaneous countermeasure conditions during the recognition of familiar items in a CIT, providing new insights into the potential of contactless, AI-assisted lie detection.

## Background

Non-instrumental methods of deception detection, which rely on behavioral cues such as facial expressions or body movements, have consistently shown low accuracy, often not exceeding chance level (around 50–55%)^11^. This limited effectiveness stems from the large number of behavioral variables and their non-specific nature, as the same changes in human behavior can appear in both deceptive and honest contexts.

Due to these limitations, instrumental methods have been developed, with the polygraph being the most prominent example. The first substantial forensic polygraph examination in a legal context took place in the United States in the 1920s, notably in the Frye case (1923), which marked a key step in the institutional recognition of psychophysiological lie detection. So far, instrumental methods have demonstrated lie-detection accuracy that substantially exceeds intuitive human judgment, particularly when standardized scoring rules and well-controlled CIT or CQT protocols are employed^12^

Before exploring the application of eye-tracking for detecting concealed knowledge, it is essential to review the psychological and physiological principles underlying the Concealed Information Test (CIT). The CIT is the most scientifically validated test for detecting concealed knowledge^13^. Its strong theoretical foundation explains how CIT-related cognitive processes elicit measurable reactions during polygraph examinations, resulting in high hit rates and a low incidence of false accusations of innocent individuals. In Japan, approximately 5,000 CIT-based polygraph examinations are conducted annually as an information gathering tool in real-life criminal investigations^14^. In other countries, such as Poland and Lithuania, the CIT can be employed either as a standalone method or in conjunction with the Comparison Question Test (CQT).

A single CIT test consists of one key element (a relevant stimulus representing a detail of the investigated crime) and several neutral or irrelevant elements. For example, if the stolen item were a gold ring, the sequence of items presented during the CIT might include earrings, a necklace, the stolen ring, a brooch, and a bracelet. An innocent person, not involved in the theft, should not react differently to the key element, as they have no prior knowledge of the crime. In contrast, for a guilty individual, the gold ring is familiar and meaningful, eliciting an orienting response (OR) that is enhanced by its rare presentation among neutral items^15^. The OR is determined by the personal significance of the relevant stimulus. Moreover, recent studies^16^ suggest that differential responses to relevant items may also result from inhibition attempts (IA) driven by motivation to conceal recognition. These attempts to suppress arousal, in order to appear innocent, can incur physiological costs, such as reduced heart rate and respiration. This differential response to the key element is referred to as the “CIT effect”^17^.

According to established guidelines, if a polygraph examination is conducted using the CIT technique alone, a minimum of four key items must be identified. These critical elements, selected from the information collected about the crime, will serve as the basis for the construction of four subtests and will be presented during the examination.

In traditional polygraph-based CIT examinations, test scoring is typically based solely on point assignment in the electrodermal channel, without evaluation of respiration or blood-pressure changes. This traditional discrimination method, proposed by Lykken, presents both benefits and drawbacks. First, this approach is practical and easy to use in field examinations; it can be applied without parameter estimation and quantification. Second, even if physiological levels vary between tests due to habituation, correction is not necessary because responses are rated within each test. The disadvantage is that this system ignores quantitative variations between answers to relevant and non-relevant stimuli. For example, the score would remain the same even if the response to the relevant stimulus was two or three times larger than the next largest response^18^.

However, a full understanding of the ’CIT effect’ requires comparing the evidence obtained using different methodological approaches. Since physiological responses to concealed information are influenced by both orienting responses and attempts to inhibit recognition, measures other than the polygraph may also serve as indicators of recognition.

Therefore, years of research on eye behaviors and cognitive processes^19^, including memory, attention, and decision-making, have provided a valuable foundation to investigate oculomotor characteristics in the detection of hidden information.

A study^20^ investigating the impact of memory on eye behavior demonstrated that gaze patterns depend on task instructions. When participants were instructed to avoid looking at a familiar face, they tended to focus more on unfamiliar faces. Conversely, when instructed to recognize the familiar face, their gaze remained longer on the familiar face. Importantly, the timing of these effects differed between tasks: in the recognition task, participants’ eyes moved almost immediately to the familiar face, showing that recognition of known stimuli occurs very quickly, whereas in the avoidance task, participants initially looked at the familiar face but gradually shifted their gaze toward the unfamiliar faces, indicating that deliberately suppressing attention to familiar stimuli is a slower, more controlled process.

Subsequently,^21^ investigated the potential impact of deception on the recognition of familiar faces using gaze behavior. They found that, compared to faces that were new to the participants, personally familiar faces elicited fewer fixations, fewer returns to previously observed regions, and less sampling of facial features during acts of deception.

Research using a traditional CIT with sequentially presented stimuli has shown that when participants view a relevant stimulus-one associated with concealed knowledge-they tend to blink less and make fewer fixations, but the duration of each fixation is longer^22^. This suggests that relevant stimuli capture attention more strongly, requiring sustained visual processing. Pupil size has been observed to increase both when participants attempt to conceal knowledge and when they reveal it^23^, indicating that pupil dilation alone cannot serve as a reliable marker of deception. Furthermore, studies have found that fixation durations become even longer when participants are trained to use countermeasures^24^, demonstrating that deliberate strategies can significantly influence gaze behavior during concealed information tasks.

Building on findings regarding fixation patterns and the CIT effect, researchers have identified an additional advantage in using ocular features within the CIT. Unlike traditional polygraph tests, eye-tracking allows for the simultaneous presentation of CIT stimuli. By defining regions of interest within these parallel stimuli, researchers can extract a range of behavioral parameters, including dwell time on the center of the screen, the proportion of fixations on relevant versus neutral images, and several other metrics^25^. Moreover, in CIT paradigms involving face recognition, the duration of gaze on specific facial regions can be quantified, providing further insight into cognitive processing and recognition during deception.

Presenting stimuli simultaneously also makes it possible to examine how specific cognitive processes influence eye movements when a person tries to conceal familiarity with a crime-related object. For example,^9^ investigated a modified CIT that included a short-term memory task. The procedure had two phases. In the encoding phase, participants were shown four faces at the same time. In the test phase, a single face was presented, and participants had to decide whether it had appeared in the previous display. The researchers hypothesized that familiar faces would attract less initial attention than new faces, because encoding familiar information requires fewer cognitive resources. Consistent with this idea, the results showed that familiar faces initially drew more direct attention due to the OR, but this was quickly followed by avoidance, suggesting that participants were attempting to hide their recognition.

Consciously directing attention toward neutral stimuli is typically associated with attempts to avoid detection. Importantly, even when participants were instructed on how to apply countermeasures, avoidance behavior remained evident^9^. When the recorded eye movement data were analyzed using a machine learning classification algorithm, the study achieved a detection accuracy of 88%, demonstrating the potential of combining eye-tracking measures with AI approaches to identify concealed knowledge.

## Methodology

This study aimed to develop a novel eye-tracking–based method for detecting concealed knowledge and to directly compare its classification performance with that of traditional polygraph examinations under identical experimental conditions. We focused on benchmarking the proposed approach against the polygraph procedures currently used in forensic practice, rather than alternative polygraph methods.

The study employed a mock crime scenario followed by the collection of physiological measurements, including eye movements and polygraph data. This design choice was motivated by evidence indicating that participants’ responses vary depending on whether they are questioned about events they personally experienced versus events they only observed or heard about. Due to the potentially sensitive nature of these procedures, the study protocol was reviewed and approved by the Ethics Committee of the AGH University of Krakow. All participants received a comprehensive explanation of the research procedures and provided their written informed consent prior to participation. All procedures were performed in accordance with the ethical standards of the institutional review board and with the 1964 Declaration of Helsinki and its later amendments. The following sections describe the participants, the experimental design, the procedures, and the data analysis methods used to develop the eye-tracking-based method.

### Participants

A total of 39 participants participated in the study, primarily students and employees of AGH University of Krakow, ranging in age from 19 to 60 years (M = 28.05, SD = 9.5). Of the participants, 56% identified as female and 44% as male. Each participant was randomly assigned to either the experimental (“guilty”) or control (“innocent”) group, resulting in 20 and 19 individuals per group, respectively. To encourage participation, participants were offered a non-monetary reward in the form of a guided visit to one of AGH’s restricted-access laboratories and the opportunity to take part in a real polygraph examination. This approach aimed at providing motivation while maintaining the ethical and methodological standards of the study.

### Study protocol

The study was conducted in two phases: a simulated crime scenario followed by a deception detection test. In the first phase, only participants assigned to the “guilty” condition engaged in the mock theft, while those in the “innocent” condition were deliberately kept naïve to the nature and sequence of the crime. In the second phase, participants underwent two separate deception detection assessments: a traditional polygraph examination and an eye-tracking measurement. All participants were instructed to maintain their assigned roles and not reveal their group designation. Those in the “guilty” group were asked to conceal their familiarity with the mock crime details to avoid detection by both systems, although no specific strategies or guidelines were provided for doing so.

Additionally, to improve the reliability and objectivity of the study outcomes, the experimental procedures involving polygraph and eye-tracking assessments were randomized with respect to the order of method administration between participants. This randomization was implemented to minimize potential order effects, such as habituation of participants.

### Mock crime scenario

In the mock crime scenario, participants were invited to the laboratory and first read written instructions explaining that they would take part in a lie detection experiment involving both an eye tracker and a polygraph. To determine their role, each participant selected one of two sealed envelopes containing instructions, which randomly assigned them to either the guilty or innocent condition. Only the experiment coordinator was aware of the assignments, ensuring that each participant’s group membership was accurately recorded.

Participants assigned to the guilty condition were required to complete a standardized mock crime task designed to establish crime-related knowledge. The scenario consisted of seven critical details and involved the following steps:

1. Put on the *black cap* and *latex gloves* provided in the instruction envelope to avoid leaving identifiable traces.
2. Obtain a *key* from the lecturer’s jacket.
3. Use the key to enter the lecturer’s office.
4. Access the lecturer’s laptop.
5. Log into the lecturer’s *Gmail* account using a password hidden in a folder marked with the *lecturer’s photograph*.
6. Locate and transcribe the *examination questions*.
7. Deposit the written copy in a *black bag* placed at a predetermined location.

The mock crime phase was estimated to last approximately 15-20 minutes per participant; therefore, participants assigned to the innocent condition, who did not perform the mock crime and were not exposed to any information relevant to the crime, were also asked to wait 15 minutes before proceeding to the test phase. This procedure prevented their immediate identification by the deception detection examiners.

### Concealed information test

The CIT was administered immediately after the mock crime procedure. The stimuli were presented as pictures and were carefully selected to ensure that crime-relevant information could be easily distinguished from neutral items. The images were also matched in terms of visual and cognitive complexity to ensure that no item was more demanding than another. The key elements were chosen based on their likelihood of being noticed and remembered by the perpetrator^26^ and are highlighted in bold in the scenario description above; however, these items were not highlighted in any way in the copy received by the participants.

To enable a direct comparison of polygraph and eye-tracking accuracy, both examinations used the same type of stimuli (relevant and neutral) and identical instructions explaining the CIT procedure. Furthermore, to mitigate any potential influence from the examination sequence, the order was altered so that half of the participants underwent the polygraph examination, followed by the eye-tracking procedure, while the remaining participants experienced the reversed sequence.

Before the CIT, participants underwent a brief pre-test interview that included questions about details of the mock crime, such as the stolen item and the perpetrator’s appearance. Following this, they received a concise explanation of the CIT procedure, emphasizing that crime-related knowledge could be used to identify guilty individuals. Participants who had committed the theft were expected to recognize specific crime details and respond differently to relevant stimuli, whereas innocent participants, lacking such knowledge, were not expected to show differential responses. Prior to each stimulus presentation, participants were informed about the focus of the upcoming CIT question (e.g., “If you committed this theft, you would recognize what was stolen”). Based on this pre-CIT briefing, it was anticipated that guilty participants might attempt to avoid detection by diverting their gaze away from relevant stimuli, consistent with previous research^20^^21^.

### Polygraph examinations

All subjects were tested regarding their participation in the mock theft by an experienced professional polygraph examiner using a computerized presentation of the CIT. This solution aims to improve the standardization of the test and reduces the likely impact of the polygraph examiner as much as possible^27^. Physiological data were recorded using the Lafayette LX4000 polygraph system, which samples all channels at up to 120 Hz. Three psychophysiological measures were collected: blood pressure, respiration, and electrodermal activity (EDA). Respiration was measured using two pneumatic assemblies to capture thoracic and abdominal changes. EDA was measured with a constant-current system (4 *μ*A, range 10 k*Ω* to 2.9 M*Ω*) using two finger electrodes. Blood pressure and pulse rate were recorded through a standard arm cuff. A seat pad movement sensor was also used to monitor participant movement. All measurements were performed in a quiet and controlled environment.

In the polygraph examinations, the CIT consisted of one block of four subtests (4-CIT). Each subtest began with a buffer item, followed by four neutral and one key element presented in one of five fixed orders. For example, if the lecturer’s photograph served as a critical stimulus, the buffer and neutral items consisted of images of unrelated individuals. Similarly, if the test exam served as a key element, the buffer and neutral items were various unrelated documents. Overall, participants were presented with four questions, each comprising six items, resulting in a total of 24 items. Prior to the CIT presentation, the examiner discussed the questions with participants but did not present the test items. Discussing CIT elements in advance can lead to habituation^28^^29^ and may increase the likelihood of false-negative outcomes.

In the polygraph examination, only the sequential CIT setup could be used. Stimuli were therefore presented centrally on the screen for six seconds each, with inter-stimulus intervals (ISI) ranging from 15 to 20 seconds. Simultaneously, participants heard an audio version of the stimuli through speakers (mean duration: 4 s for the question, 1 s for each item), generated using the LXSoftware speech synthesis system. During question presentation, the screen remained blank. Participants were instructed to repeat the names of the items aloud as they were presented. Notably, participants did not provide a “no” response to every presented item, which meant that a guilty examinee did not need to lie when confronted with the key items of the tests. Upon completion of the examination, participants were not informed of the polygraph results.

When the examination was over, participants were not informed about the results of the polygraph examination.

### Eye tracker examination

**Fig. 1:**
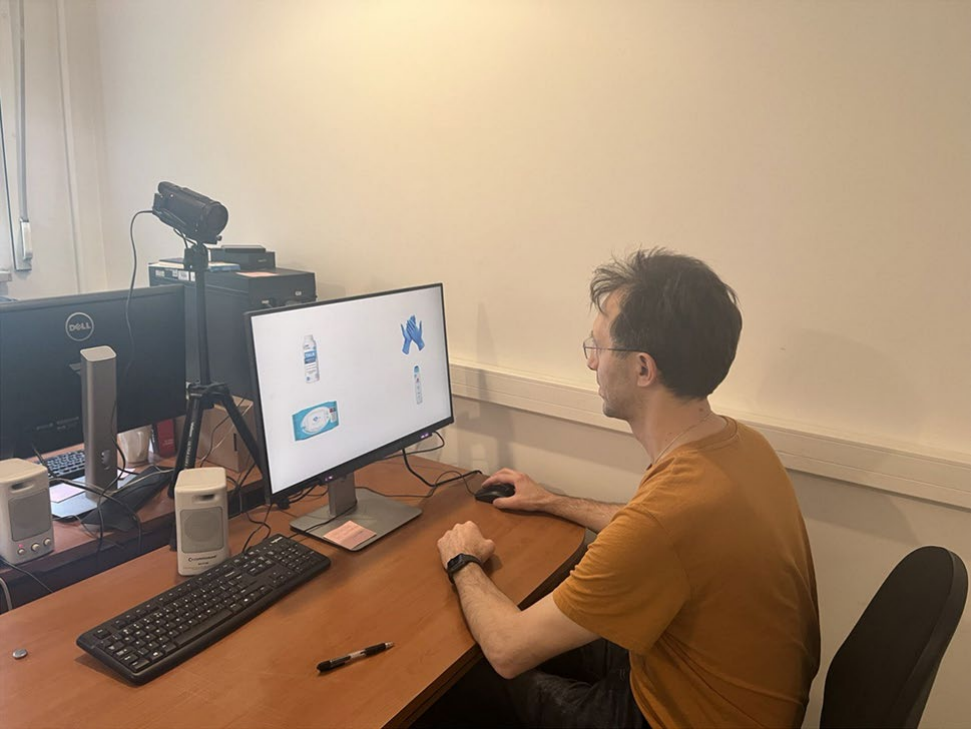
Experimental eye-tracking-based CIT test setup.

Both the setup and methodology of CIT using an eye-tracker differed from that employed with the polygraph. Unlike the polygraph, the eye-tracking setup was much simpler and did not require any sensors to be attached to the participants’ bodies. Additionally, this method allows the presentation of multiple stimuli simultaneously, enabling the assessment of additional features grounded in both orienting and inhibition theories. Participants were simply seated in front of a computer monitor and their eye movements were recorded using the Tobii Pro Fusion desktop eye tracker and the Tobii Pro Lab software. Visual stimuli were displayed on a 27-inch full HD monitor, positioned approximately 70 cm from the participant. The eye tracker was placed below the computer screen to ensure an unobstructed view for the participants. The overall test configuration is illustrated in Fig. 1. To ensure experimental consistency, all sessions were conducted under controlled and constant lighting conditions: window blinds were closed to eliminate natural light variations, and the same artificial lighting setup was used for all participants.

Before recording the eye-tracking signal for each participant, a brief calibration procedure was conducted, during which participants were asked to follow points that appeared on the screen with their gaze. Once calibration was complete, the eye-tracking examination began. During this stage, participants were presented with seven questions, each followed by the simultaneous display of four stimuli - one critical stimulus and three neutral stimuli. The visual stimuli used in the eye-tracking tests varied in complexity and cognitive demand: some containing text that required reading and others consisting of simple images that engaged visual perception. Single-object stimuli with low complexity included items such as a cap or a key, whereas more complex stimuli involved the document that was stole in the mock crime scenario. In addition, one of the tests featured stimuli in a form of the image of the victim’s face. This design aligns with previous findings that emphasized that eye movements and attentional allocation are influenced not only by visual properties but also by the semantic and task relevance of stimuli^30^.

**Fig. 2:**
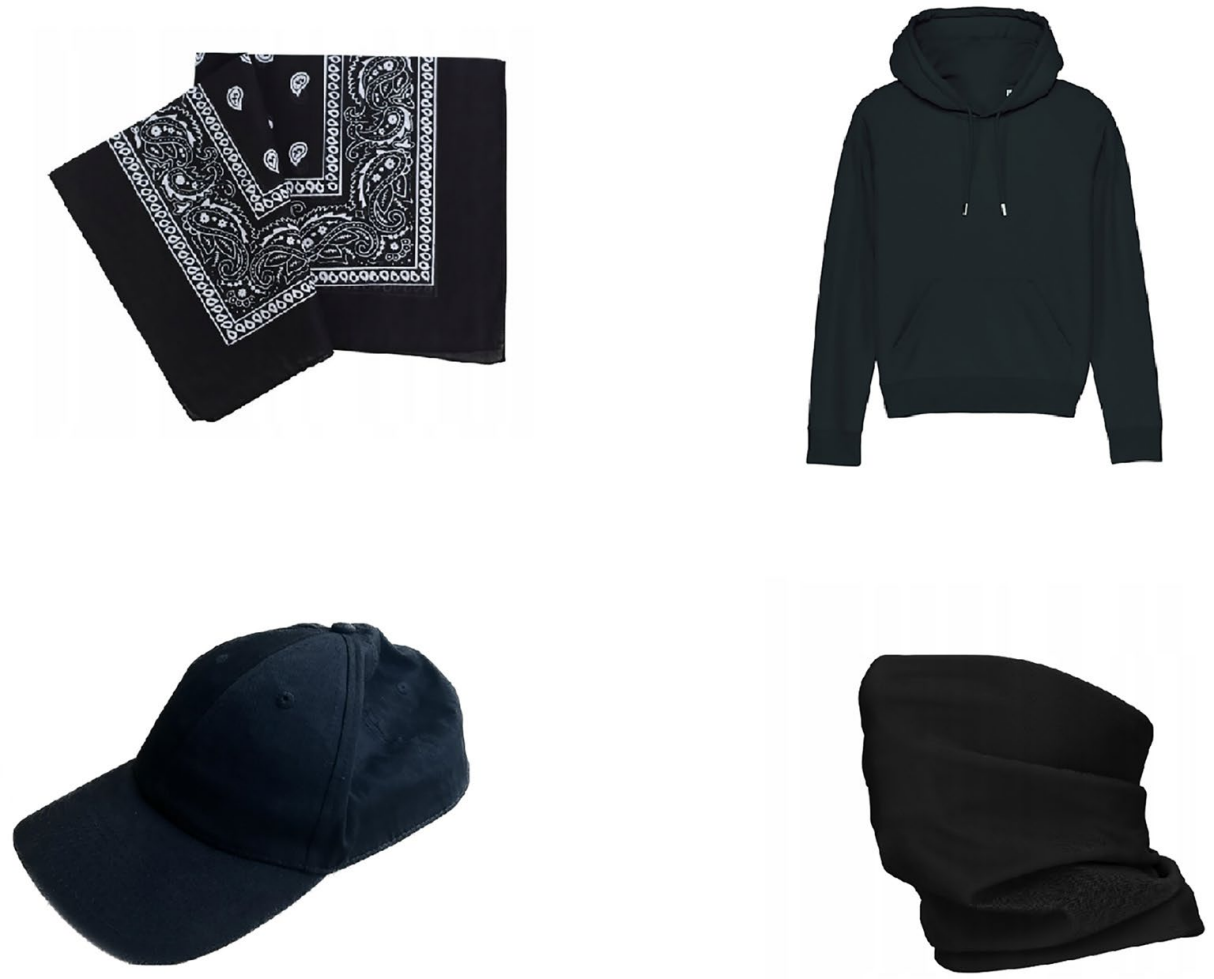
An example slide presenting stimuli shown to the participant after asking question number 1.

The content of the questions and the corresponding stimuli are further detailed in Table 1, while example slides showing the four stimuli presented to participants in one of the tests are provided in Fig. 2. Each slide was displayed for 7.5 seconds, which was sufficient for the participants to visually inspect all four stimuli, consistent with standard practices in such tests. This cycle was repeated three times, with the position of the stimuli changed in each repetition to control for position effects.

To ensure consistent testing conditions for all participants, the questioning process was fully automated using the HeyGen video generator. This tool allowed for the conversion of text into video, with a selected avatar serving as the speaker, providing standardized delivery of all questions. The questions were prepared so that each was presented for a fixed duration of six seconds. Following each question and before the presentation of the stimuli, a fixation cross appeared at the center of the screen to direct the gaze of the participants to a consistent starting point for each slide, ensuring uniform visual attention throughout the trials.

**Table 1:** Questions and stimuli used in eye-tracking based CIT (critical stimulus in bold).

Question	Stimuli
Do you know what the intruder used to conceal their identity	hoodie, bandana, skiing mask, *cap*
Do you know what the perpetrator used to make identification difficult?	deodorant, baby powder, wet wipes, *gloves*
Do you know what the perpetrator used to open the door?	lockpick, magnetic chip, code, *key*
Do you know to whom the bulgarized office belonged?	3 faces not related to the theft, *lecturer’s face*
Do you know which email was logged into?	outlook, interia, onet, *gmail*
Do you know what was stolen?	directive, grades, measurements, *exam*
Do you know where the stolen item was hidden?	box, mailbox, backpack, *black bag*

### Post study questionnaires

After completing both examinations, participants’ motivation to follow instructions was assessed using a six-point scale. For the guilty group, the scale measured motivation to conceal recognition of key items, whereas for the innocent group, it assessed motivation to demonstrate their innocence by cooperating with the examiner (1 = not motivated at all, 6 = very motivated). The mean motivation scores were 5.05 for the guilty group and 5.0 for the control group. This slight numerical difference was not statistically significant, indicating minimal variation in the subjective perception of engagement between the two groups.

## Data processing and analysis

This section outlines the methods used to preprocess, quantify and analyze the data obtained from both polygraph and eye-tracking assessments.

### Polygraph examination

To maintain the principle of blind scoring, all CIT charts were evaluated by an additional independent polygraph examiner who was not present during the examination and received only the physiological signals recorded for analysis. This examiner was also unaware of the group assignments of the examinees. Before feature extraction, a global inspection of all charts was conducted to identify and remove artifacts.

As authoritative sources on the CIT^1^ recommend, the Lykken scoring method was applied for test data analysis to classify the participants as guilty or innocent. Many studies so far have applied this scoring system to measure the CIT accuracy^31,32^. In this system, only the electrodermal activity channel (EDA) is evaluated, based on diagnostic responses that occur within the onset window. Electrodermal responses were quantified as the maximal increase in conductance from stimulus onset to five seconds post-onset. Responses to the first stimulus in each subtest were excluded, as these served solely as buffer items to absorb initial arousal. During test data analysis, EDA response amplitudes were ranked from highest to lowest for each stimulus within a subtest. Lykken’s method converts the ranks into integer scores as follows:

- +2: Assigned if the key stimulus elicits the highest response in the subtest.
- +1: Assigned if the key stimulus elicits the second highest response.
- 0: Assigned if there is no difference in amplitude between the relevant and irrelevant stimuli.

Four subtest scores were then summed to calculate a total test score, which was compared to a predefined threshold to yield a binary classification: “Recognition Indicated” or “No Recognition Indicated.” In this study, a total score of +5 or higher indicated recognition of crime-related details, classifying the participant as guilty, while a total score +4 or below indicated an innocent outcome.

### Eye tracker examination

Prior to analysis, the acquired eye-tracking data was pre-processed using Tobii Pro Lab software. The recorded eye movement signals were segmented into time-of-interest (TOI) and area-of-interest (AOI) segments. Each TOI corresponded to the duration during which a single slide containing stimuli was presented, while each AOI represented a specific region of the slide containing one stimulus, effectively dividing each slide into four distinct areas. After this segmentation, relevant eye-tracking features were extracted from the preprocessed signals for subsequent analysis.

Tobii Pro Lab is capable of extracting a wide range of eye-tracking features; however, only a subset was selected for analysis in the present study. For each of the four objects presented on a slide, a feature vector was constructed. This vector included the average pupil diameter, along with summary statistics of gaze behavior derived from Tobii Pro Lab. Specifically, for each of the three gaze metrics (fixations, visits, and glances), we computed the total, mean, minimum, and maximum durations, as well as the number of occurrences and the duration of the first event. These gaze metrics are defined as follows:

- *Fixations*: Intervals during which the eyes remain relatively stationary, allowing central foveal vision to acquire detailed information about the observed object. In Tobii Pro Lab, a fixation is identified as a sequence of raw gaze points whose estimated velocity falls below the threshold specified by the I-VT gaze filter.
- *Visits*: The period from the onset of the first fixation on an AOI to the offset of the last fixation within that AOI, excluding entry and exit saccades.
- *Glances*: The interval from the saccade leading into the AOI to the last fixation on that AOI, excluding only the exit saccade.

Although fixations, visits, and glances all derive from the same underlying gaze signal, each emphasizes different aspects of visual behavior and provides complementary information for classification. Fixations capture detailed moments of visual attention, visits reflect overall engagement with an AOI, and glances represent the transitions into an AOI. By combining these measures, richer features can be extracted from the gaze data, enhancing the performance of classification models.

**Fig. 3:**
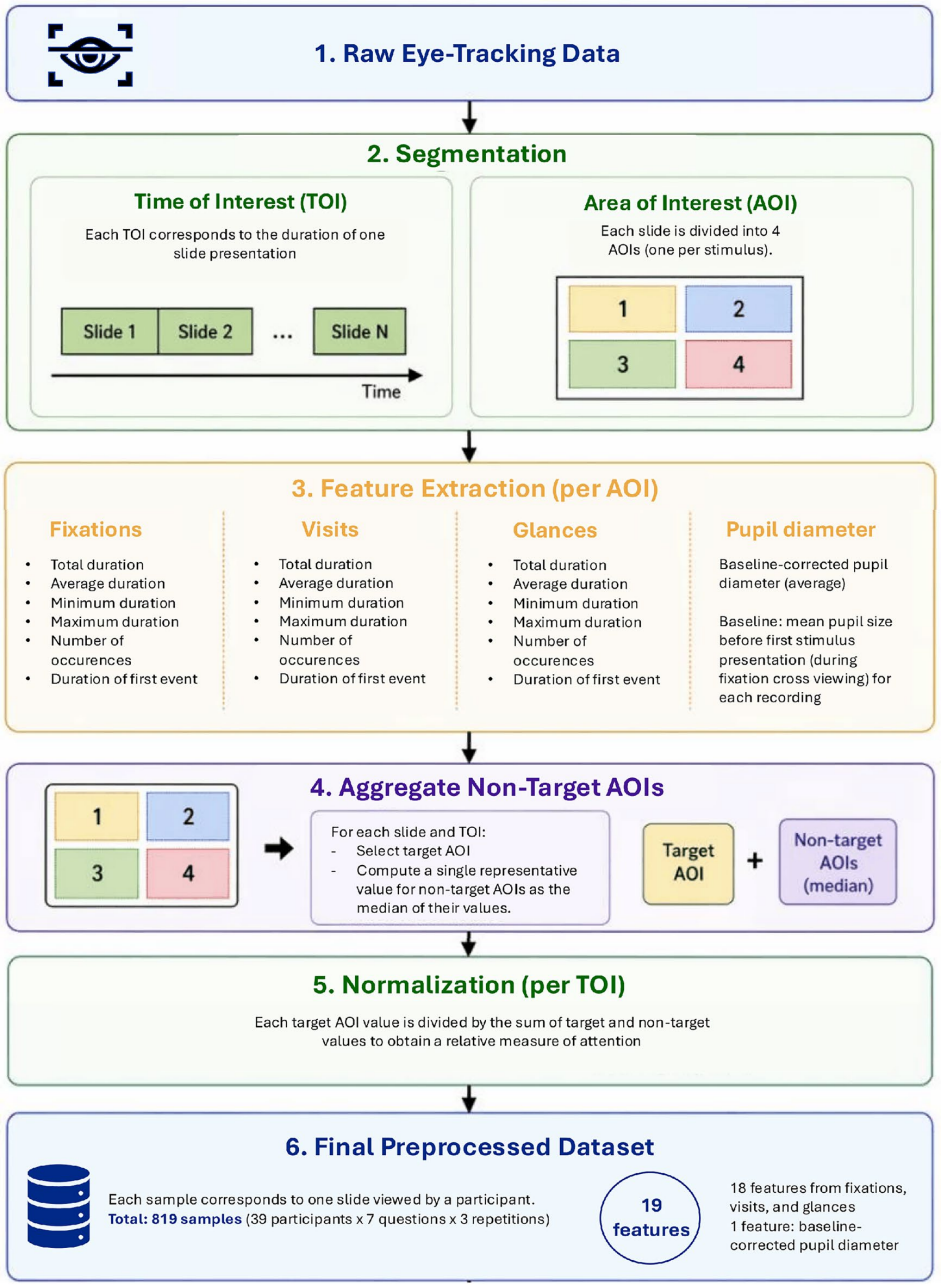
Eye-tracking data preprocessing pipeline.

Each sample in the full dataset, constructed from all recorded signals, corresponds to a single slide viewed by a participant, resulting in a total of 819 samples (39 participants *×* 7 questions *×* 3 repetitions). The feature vector for each sample included values for the target AOIs, as well as a single representative value for the non-target AOIs, computed as the median of their values. To account for differences in overall gaze behavior across participants, each target-AOI value was normalized by dividing it by the sum of the corresponding critical and non-critical values within the same time of interest (TOI). This procedure provides a relative measure of attention directed toward target stimulus while controlling for general viewing patterns. In this way, we aimed to reduce the dimensionality of the feature space, simplifying the dataset while retaining essential information about participants’ viewing behavior. For each AOI, the features captured total duration, average duration, minimum and maximum duration, number of occurrences, and duration of the first event, computed separately for fixations, visits, and glances. In addition, one feature represented average value of baseline-corrected pupil diameter. Baseline correction was performed on a per-recording basis using the mean pupil size measured before the initial stimulus presentation (during the first fixation cross). This approach resulted in a final vector of 19 eye-tracking features per sample after normalization. The complete preprocessing pipeline is shown in Fig. 3.

The resulting feature matrix, together with the target vector (a binary label indicating whether a participant was guilty or not), was initially used in its complete form to train three classifiers: a support vector machine (SVM) with an RBF kernel, extreme gradient boosting (XGBoost), and a random forest classifier. Subsequently, the training procedure was repeated with feature selection applied within each cross-validation fold using a univariate filter-based approach, in which features were ranked according to their ANOVA F-statistic reflecting between-class discriminatory power, and only the top-ranked features were retained for model training. The number of features retained (k) was treated as a hyperparameter and optimized for each hyperparameters set in the grid to maximize classification performance. All three selected classification algorithms are capable of handling high-dimensional, potentially correlated features and capturing complex, nonlinear relationships. Each classifier also includes mechanisms to reduce overfitting - XGBoost through regularization, random forest through ensemble averaging, and SVM via margin maximization - making them well-suited for datasets with relatively small sample sizes.

Additional ablation studies were then performed using only the classifiers that achieved the best overall performance across all stimuli, in order to evaluate whether removing specific types of stimuli would improve classification results. The stimuli were categorized into four types: face stimuli (the lecturer’s face), simple stimuli (objects such as gloves, bag, keys, and cap), mixed stimuli (combinations of text and images - email logo), and text stimuli (exam-related items). By selectively excluding one category at a time, we assessed the contribution of each stimulus type to the overall classification performance.

As the selection of model hyperparameters can substantially influence classification accuracy, all models were optimized via a grid search over a range of values. The full range of the hyperparameters used is provided in Table 2.

**Table 2:** Hyperparameter grid used for model optimization.

Model	Category	Hyperparameter	Tested values
SVM	Regularization	gamma	0.0001, 0.001, 0.01, **0.1**, 1, 10
		C	**0.001**, 0.01, 0.1, 1, 10, 100
Random forest	Tree complexity	Max depth	3, 5, 7, None
		Min samples split	2, 5, 10
	Leaf size	Min samples leaf	1, 2, 4
		Max features	sqrt, log2, None
	Boosting/number of trees	Number of estimators	100, 200, 300
XGBoost	Tree complexity	Max depth	3, 4, 5, 6
		Min child weight	1, 3, 5
	Boosting	Number of estimators	50, 100, 150
		Learning rate	0.05, 0.1, 0.2
	Feature subsampling	Subsample	0.7, 0.8, 1.0
		Column sample by tree	0.7, 0.8, 1.0
	Regularization	L1 regularization	0, 0.1, 0.5, 1
		L2 regularization	1, 2, 5
	Split threshold	Gamma	0, 0.1, 0.2

Bold value indicates statistically significant.

Given the relatively small size of the dataset, we employed a GroupKFold cross-validation strategy to assess model performance, using two participants as a test set in each fold. We opted for two-groups-out cross-validation to obtain a reliable estimate of our model’s ability to generalize to unseen participants instead of one participant out because each participant belongs entirely to a single class, leaving only one participant in the test set could result in the model always predicting that participant’s class, yielding misleading, overly optimistic performance metrics. By holding out two participants in each test fold and ensuring both classes are represented, we can identify cases where the model might otherwise favor a single class, thereby avoiding inflated or misleading performance metrics. In contrast, traditional GroupKFold cross-validation with 5 or 10 folds can be problematic for small datasets: withholding even a single fold may remove a substantial portion of the already limited data, leaving training sets too small to learn meaningful patterns and producing unstable performance estimates. Our two-groups-out strategy addresses this limitation by retaining larger, more informative training sets while still ensuring fully independent, participant-level evaluation, resulting in more reliable and realistic assessments of model generalization.

For each combination of hyperparameters the predictions were aggregated at the participant level and a participant-level scoring function was applied to quantify the performance of the model.

## Results

### Polygraph examination

The process of chart evaluation starts with global analysis aimed at assessing the overall quality of the recorded data. During this phase, the examiner detected the use of physical countermeasures by some participants, manifested as deep breathing or finger and hand movements observable in certain physiological traces. Based on this visual analysis of the CIT data, 5 examinations were excluded from further numerical analysis, of which 3 belonged to the guilty group and 2 to the innocent group. Thus, only 34 of the original 39 polygraph examinations were assessed using the Lykken scoring system.

**Fig. 4:**
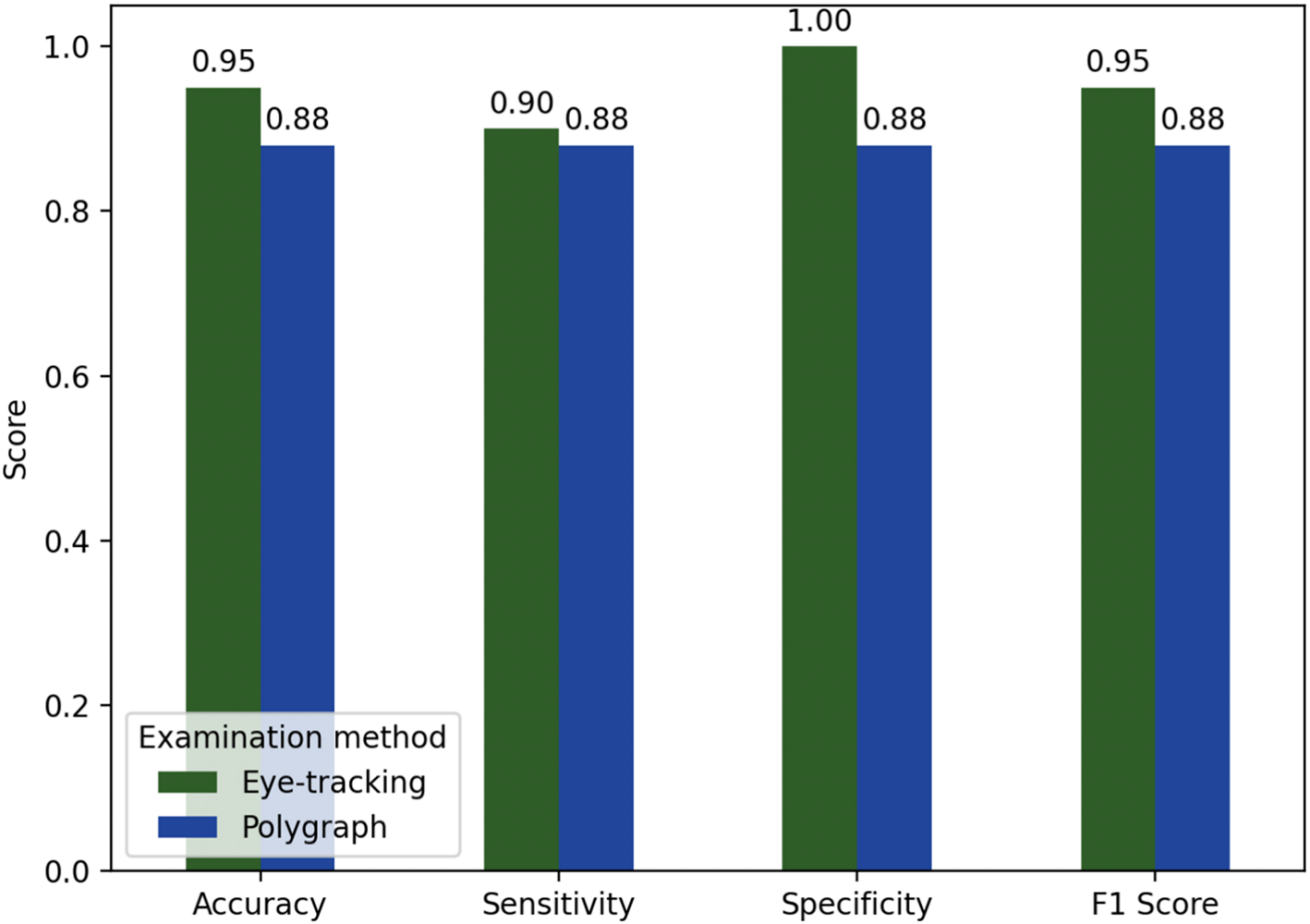
Comparison between eye-tracking and polygraph classification scores.

Results indicated that 15 of the 17 innocent examinees were correctly classified as innocent, whereas two were incorrectly identified as possessing concealed crime knowledge. Similarly, 15 of the 17 guilty examinees were correctly classified, while two were incorrectly classified as innocent. Consequently, the polygraph classification yielded identical values for Accuracy, Sensitivity, Specificity, and F1-score, all equal to 0.88 (Fig. 4). This symmetry reflects the underlying confusion matrix, in which 15 of 17 cases were correctly classified in each group, resulting in two misclassifications per group.

### Eye tracker examination

None of the recordings had to be excluded from the analysis, ensuring that the full dataset of participants was available for model training and evaluation.

The classification evaluation was initially conducted using the full set of extracted features, without feature selection, and was subsequently performed with feature selection applied within each cross-validation fold. For the best-performing classifier, which used a feature subset of 19 eye-tracking variables, feature stability was examined across cross-validation folds. Seven features were selected in all folds, indicating high robustness and consistent contribution to classification performance. These features were: Total duration of fixations, Total duration of glances, Total duration of visits, Number of fixations, Number of glances, Number of visits, and Average pupil diameter (baseline-corrected). The normality of these features was assessed and indicated non-normal distributions; therefore, non-parametric Mann–Whitney U tests were used to examine group differences. The results showed that all seven features differed significantly between the groups. Additionally, descriptive statistics for the selected eye-tracking features are reported in Table 3 and are presented at the group level. The interquartile ranges indicated substantial variability in gaze behaviour across participants, with strong overlap between groups. However, consistent shifts in medians toward higher values in the guilty group suggest systematic differences in both ocular and temporal features despite heterogeneous distributions. It is important to note, that individual samples corresponding to each question and time interval were fed into the model separately, allowing the classifier to learn from trial-level dynamics as well as aggregate participant-level patterns. This procedure led to performance improvements across all evaluated models. The best-performing classifier, the SVM with an RBF kernel, correctly classified 37 out of 39 participants, corresponding to an accuracy of approximately 94.9%. The classification results obtained before and after feature selection are reported in Table 4.

**Table 3:** Descriptive statistics (median [Q1–Q3]) of eye-tracking features for control and guilty groups.

Feature	Control	Guilty
Total duration of fixations	508.0 (135–1196)	800.0 (331–1408)
Total duration of glances	660.0 (164–1429)	1026.0 (428–1732)
Total duration of visits	580.0 (135–1329)	935.0 (392–1620)
Number of fixations	2.0 (1–4)	3.0 (2–5)
Number of glances	1.0 (1–2)	2.0 (1–3)
Number of visits	1.0 (1–2)	2.0 (1–3)
Average pupil diameter (baseline corrected)	−0.403 (−0.755-0.000)	−0.434 (−0.726−(−0.116))

**Table 4:** Classification performance metrics for all tested classifiers. Accuracy, sensitivity, specificity, and F1-score are reported for each model.

Training configuration	Classifier	Accuracy	Sensitivity	Specificity	F1-score	ROC AUC
Without feature selection	SVM (RBF)	0.90	0.80	1.00	0.89	0.90
	Random forest	0.65	0.90	0.40	0.72	0.62
	XGBoost	0.72	0.90	0.53	0.77	0.67
With feature selection	**SVM (RBF)**	**0.95**	**0.90**	**1.00**	**0.95**	**1.00**
	Random forest	0.64	0.90	0.40	0.72	0.62
	XGBoost	0.74	1.0	0.5	0.67	0.75

Bold value indicates statistically significant.

In conducted ablation studies in which different types of stimuli were selectively excluded to evaluate their contribution to classification performance, the accuracy remained largely unchanged across the various exclusion conditions, with the exception of the model excluding simple stimuli (e.g., gloves, bag, keys, cap), which showed a slight decrease in classification accuracy (from 94.9% to 87.2%). This suggests that while most stimulus types contribute similarly, simple stimuli provide unique information that enhances classifier performance.

## Discussion

### Building on established research foundations

Previous studies have provided valuable theoretical insights into memory, attention, and deception detection via ocular features. Although these investigations differ in specific aspects, such as the oculomotor features recorded or the types of stimuli utilized, the majority employed the conventional sequential presentation of test stimuli in CIT. Yet, they did not achieve a notable advancement in the detection of concealed knowledge^22^^24^. Generally, existing research has yielded important findings with two key conclusions for future forensic applications. First, the detection of concealed memory through eye movement is influenced by task demands^10^. Second, presenting test stimuli simultaneously may inherently improve the distinction in gaze behavior observed between guilty and innocent subjects^9^. Introducing high task demands (particularly heavy short-term memory load) substantially hinders participants’ ability to implement deceptive tactics. By taxing cognitive resources with a concurrent memory task, recognition “leakage” becomes far more evident, resulting in markedly improved detection rates. In essence, these studies confirm that elevating cognitive load is a powerful approach to counter countermeasures in CIT and reveal concealed knowledge via eye-tracking.

Importantly, the traditional CIT has proved its high accuracy in a number of laboratory studies using mock-crime scenarios. While field studies evaluating CIT in real-life settings are difficult to conduct, only a few laboratory experiments have implemented conditions that closely resemble an actual crime and lie-detection examination, and these studies have reported higher CIT accuracy (effect size = 2.09) compared to experiments conducted under less realistic conditions^33^.

Building on these findings, the CIT format has already been implemented in research using eye-tracking methodology, including paradigms that involve simultaneous stimulus presentation. Previous research has demonstrated that even in more complex CIT variants-featuring simultaneous displays of stimuli combined with additional cognitive tasks (e.g., short-term memory or visual detection tasks)-high detection accuracy can be achieved through the analysis of oculomotor responses.

These earlier multi-task CIT designs served as inspiration for the development of a new, simplified test format. In the present variant, each visual display consists of exactly four photographs: one critical (key) stimulus and three non-critical (irrelevant) stimuli, without additional cognitive requirements. The core of this protocol relies on a carefully formulated test instruction that directs participants to freely view the images, combined with time pressure during which they are required to familiarize themselves with the test stimuli within a strictly limited time window, without providing explicit verbal or manual responses.

Additionally, the objective of the present study was to complement and extend the existing knowledge of the Concealed Information Test (CIT) in eye-tracking examinations, particularly under conditions that simulate a real-life crime scenario. To this end, the study introduced several novel modifications to the CIT procedure.

The simulated theft scenario was designed to resemble a real crime. Based on prior empirical evidence^34^ indicating that different crime details vary in relevance to perpetrators: information about actions or objects that captured participants’ attention being more readily recalled, as opposed to peripheral details that tend to be less remembered, the CIT questions in our study were concentrated on central elements rather than peripheral ones. This approach was intended to enhance the likelihood that participants would form robust memories of crucial elements. Additionally, constructing questions around guilty actions (e.g., “If you committed this crime, you would recognize the stolen item”) may further enhance detection accuracy.

Among the key stimuli selected in the present study were details of crime related to the actions of the participant (e.g., how the individual entered the lecturer’s room or what they wore to avoid identification) and details that could only be observed by committing theft (e.g., the appearance of the lecturer or the specific test that was stolen). To provide a more realistic context, polygraph examinations were conducted using a real polygraph rather than the laboratory devices employed in previous studies. This gave participants the genuine experience of undergoing a forensic procedure similar to those used by police when testing suspects of crime.

Additionally, pre-test interviews and CIT instructions were incorporated to create more realistic experimental conditions. More realistic experimental conditions appear to influence the effectiveness of crime memory detection. Polygraph recordings demonstrated that relevant stimuli elicited stronger responses, similar to what would be expected in real-life scenarios, suggesting that maintaining realistic experimental conditions may be beneficial in future studies.Together, these methodological choices were designed to maximize ecological validity of this study.

### Practical comparison of eye-tracking and polygraph

According to the primary objective of this study was to investigate whether combining CIT examination with oculomotor measures and machine learning could yield results equivalent to or greater than polygraph-based CIT. In the current study, these various measurement methods demonstrated no statistically significant differences in classification accuracy; however, five polygraph examinations were excluded from the evaluation due to insufficient data quality, while no participants were excluded from the eye-tracking-based method. It is essential to recognize that this research constitutes preliminary investigations based on a limited sample of participants. Therefore, for a comprehensive assessment of the feasibility of introducing eye-tracking-based CITs, broader studies on a larger group of participants are crucial. In the final evaluation stages, these studies should also be conducted under field conditions to ensure ecological validity and robustness of findings.

**Fig. 5:**
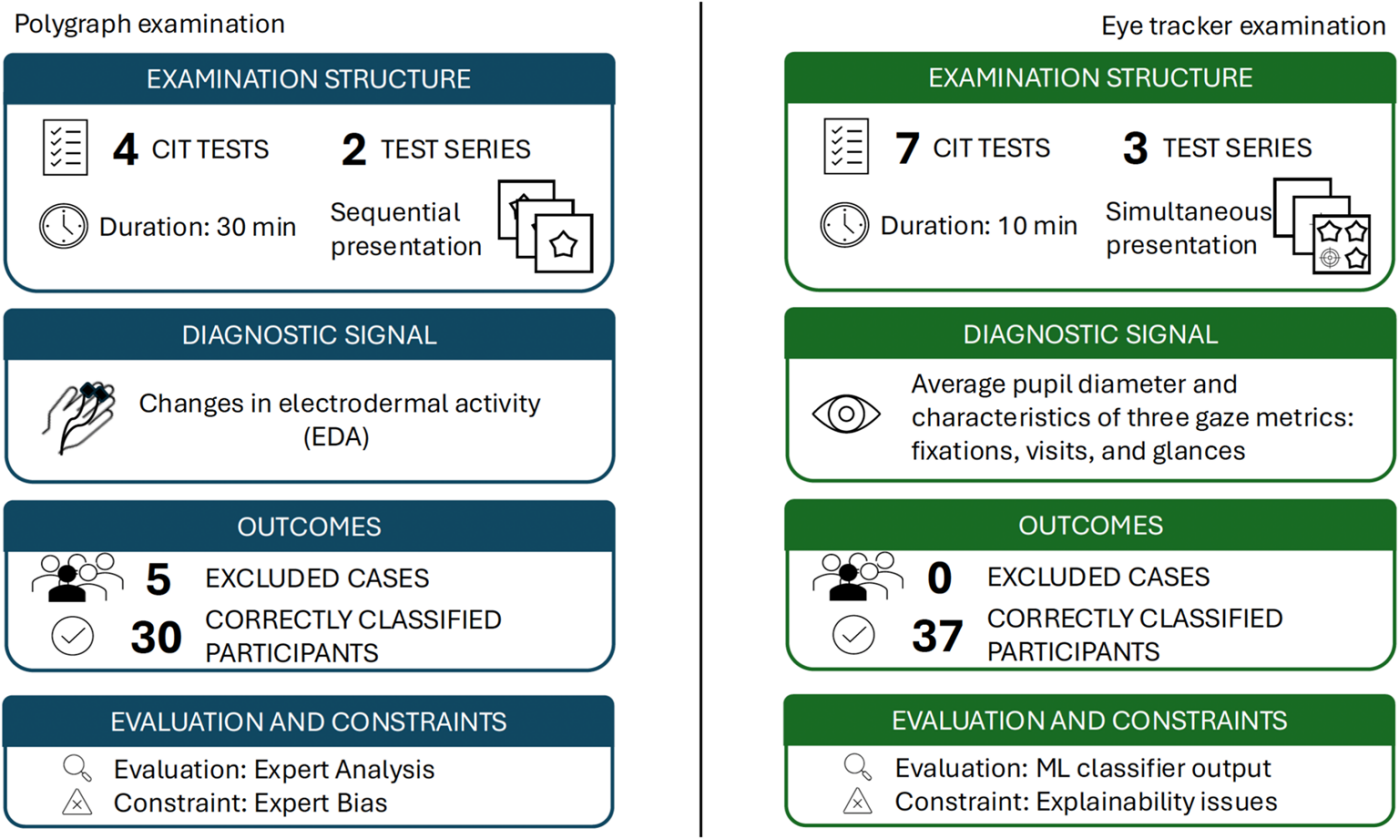
Comparison between eye-tracking and polygraph variants of CIT.

Our results indicate that while the eye-tracking-based deception detection procedure achieves accuracy similar to the traditional polygraph among participants whose recordings could be evaluated (Fig. 4), it shows a clear observable practical advantage in other aspects (Fig. 5). Most importantly, while the two methods showed similar classification accuracy, the polygraph-based approach produced conclusive outcomes for fewer participants, whereas the eye-tracking method enabled analysis of a larger number of recordings.

Furthermore, the eye-tracking examination proved more efficient, with the measurement phase lasting only 10 minutes compared to 30 minutes for the polygraph, thanks to a simultaneous presentation format that supported a more time-effective and robust CIT protocol. This shorter duration did not compromise the diagnostic value; in fact, it enabled the inclusion of more CIT tests (7 vs. 4) and additional series (3 vs. 2), while keeping the stimulus intervals shorter (7 s vs. 20 s).

The eye-tracking approach also enhances objectivity and reproducibility by focusing on well-defined oculomotor measures, which characterize fixations, visits, and glances, rather than depending solely on electrodermal activity, as in traditional polygraph-based CIT. Additionally, decision-making is driven by a machine learning classifier, minimizing expert bias, a limitation often observed in polygraph evaluations that depend on subjective judgments.

Moreover, the noninvasive and contactless nature of eye-tracking makes the method significantly more comfortable for participants, eliminating the need for physical sensors and reducing stress-induced physiological artifacts. This feature improves both data quality and ethical acceptability, especially in populations where stress reactivity may be variable or increased.

Another crucial advantage is its resistance to countermeasures. In contrast to the polygraph, which is susceptible to manipulation through the control of respiration or muscle activity, eye-movement patterns exhibit a substantially lower susceptibility to conscious regulation, thereby enhancing the method’s reliability in practical applications.

### Comparison with prior research: robust performance of the simplified CIT

Importantly, the effectiveness of the newly proposed CIT was comparable to that reported by^9^, which incorporated an additional short-term memory task. Notably, using a mock crime scenario combined with a simplified test structure did not compromise the accuracy of guilty detection in the eye-tracking procedure. These findings have practical implications for forensic applications. Consistent with previous research, they indicate that oculomotor responses differ when individuals withhold information about crime details, allowing for correct identification with an accuracy of 92.3%. Furthermore, our results suggest that the principles of oculo-based CIT construction can be simpler and easier to implement than those used in prior studies^9^. In contrast to designs that include additional tasks-which may be time-consuming and complex to develop- our approach is streamlined, with each test comprising only three neutral and one key stimulus, making it both efficient and practical for applied settings.

Finally, while other automated eye analysis solutions-such as those based on reading behavior-are already commercially available^35^, the new eye-tracking-based CIT method offers strong potential for automation and scalability, facilitating its deployment in various applied contexts like border control, security screenings, or personnel selection, where rapid, repeatable, and unbiased assessments are essential. It also aligns with broader trends in cognitive science and behavioral analytics, representing a modern, data-driven approach to deception detection that addresses long-standing limitations of traditional physiological methods.

### Forensic implications and need for further validation

While exploring the potential benefits of eye-tracking in lie detection, it is important to recognize the challenges and considerations associated with introducing new methodologies into forensic science. The objectivity and efficacy of automated lie detection systems should not eclipse the skepticism regarding the possible displacement of expert analysis within the forensic field. The incorporation of automated tools, underpinned by machine learning algorithms, prompts inquiries into the prospective function of human experts in forensic science.

In traditional polygraph examinations, the examiner is responsible for both conducting the test and making the final determination. In contrast, the introduction of automated eye-tracking analysis raises important questions about accountability for false negative (FN) and false positive (FP) errors in cases of misinterpretation by the algorithm. In addition, potential biases in the training data must be considered, as they can further increase the risks inherent in automated decision making.

Unlike polygraph examiners who can clearly explain the reasoning behind their opinion, machine learning tools often operate as “black boxes,” making it difficult to understand the basis behind their results. This lack of transparency raises doubts about the admissibility of automated evidence analysis in legal settings. Consequently, further research aimed at improving the explainability of the results is crucial.

In the field of credibility assessment, the optimal approach appears to lie in balancing innovative technologies with human expertise. Integrating forensic knowledge with automated methods can improve overall effectiveness. For example, polygraph examiners can contribute valuable experience in selecting crime-relevant and neutral stimuli, as well as providing a contextual interpretation of the oculomotor test results, thus improving the practical utility of automated systems in security applications.

Due to automatic measurement and data analysis, the new oculo-CIT-based system, in the future, after standardization of the method and its forensic validation, could serve as a tool to support police investigative work by helping to differentiate individuals who conceal crime-related knowledge from those who do not recognize critical information. The most optimal scenario would be for such a system to be applied at the initial stage of police proceedings. At that point,it could help to quickly narrow down the group of persons who may be connected to the crime and guide law enforcement actions toward individuals whose results indicate they possess knowledge about the event. However, the results of such examinations could never serve as the sole evidence of anyone’s guilt.

## Limitations

A key limitation of the present study is that the differences between the polygraph-based and eye-tracking-based CITs were not confined to the measurement modality alone, but were also confounded by variations in stimulus presentation format (sequential vs. parallel). This factor complicates direct attribution of performance differences solely to the physiological techniques employed. Consequently, the observed differences in performance of both CITs can not be attributed unambiguously to modality alone, as they may partly reflect differences in cognitive processing induced by the presentation format or interactions between these factors. Sequential presentation, used in polygraph-based CITs, likely emphasizes temporal processing, expectancy, and memory-based comparisons, whereas simultaneous stimuli presentation employed in eye-tracking paradigms, facilitates spatial comparisons and selective allocation of visual attention. As a result, although stimulus content was matched across methods, these two CIT formats may have engaged partially distinct cognitive mechanisms, which limits the direct comparability of effect magnitudes across modalities.

When interpreting differences between investigated methods, caution is warranted, and claims of absolute superiority based solely on its accuracy should be avoided. Instead, the current findings primarily demonstrate that each method is effective within its own experimental framework. Notably, the eye-tracking-based procedure offers clear practical advantages: achieved classification accuracy, required less time to administer, and was less influenced by examiness compared to the polygraph. These strengths highlight the potential of eye-tracking as a next-generation tool for both research and applied lie detection settings.

Prior to entering forensic practice, the major scientific interest should be focused on the sensitivity of eye tracker tests to countermeasures, its external validity and accuracy, as well as the specificity to deception. Firstly, one could argue that simulation-based research has insufficient external validity because experimental factors affecting participants’ perception and memory might differ from those from realistic setups. Moreover, in CIT laboratory research participants learn the pre-defined key items from instruction and are typically selected to determine their relevance and memorability that is impossible in realistic context. In addition, current research assessed the vulnerability of recognition markers under spontaneous countermeasure instructions to look at both familiar and unfamiliar items. Subsequent studies should verify the sensitivity CIT procedure presented here by providing examinees with instructions on how to use countermeasures. Finally, before applying the eye tracker-based CIT to actual practice, it is necessary to verify the present findings using a field dataset.

## Conclusion

In conclusion, this study aimed to evaluate the discriminative power of a novel eye-tracking-based concealed knowledge test in comparison to polygraph examination within the same experimental framework. This method involved extracting features from eye-tracking data corresponding to participants’ responses to critical and control stimuli. Subsequently, these features underwent classification using various machine learning classifiers through a grid search procedure to optimize parameters. The optimal classifier correctly identified 37 out of 39 participants, yielding an accuracy of 94.9%, thereby slighlty outperforming the polygraph examination, which correctly classified 30 out of 34 participants and achieved an accuracy of 88.2%. These results suggest that eye-tracking-based methods could be further explored as a potential alternative to polygraph examinations. However, it’s important to note several limitations of this research. Firstly, the sample size was relatively small, and secondly, the study was conducted in a laboratory setting with participants who faced no real consequences based on the test outcomes. Future work should also investigate important factors which may influence the sensitivity of eye tracker examination with CIT (e.g. individual psychological features, eye conditions, and countermeasures).

## Data Availability

We have made the anonymized raw eye-tracking data publicly available under DOI: 10.5281/zenodo.18525952. Regarding the polygraph data, these recordings are in a proprietary format that requires manufacturer-specific software to access and display. Moreover, the interpretation of the polygraph signals was performed manually by an expert rather than automatically, meaning the raw signals cannot be independently reused to reproduce the expert’s conclusions. Consequently, we do not provide the raw polygraph data.
